# “Not me!” a qualitative, vignette-based study of nurses’ and physicians’ reactions to spiritual distress on neuro-oncological units

**DOI:** 10.1007/s00520-024-08704-y

**Published:** 2024-07-10

**Authors:** Daniela Völz, Reinhard Grabenweger, Megan C. Best, Peter Hau, Kate F. Jones, Ralf Linker, Piret Paal, Elisabeth Bumes

**Affiliations:** 1 Department of Neurology and Wilhelm Sander-NeuroOncology Unit, Regensburg University Hospital, Regensburg, Germany; 2 Institute of Nursing Science and Practice, Paracelsus Medical University, Salzburg, Austria; 3 Institute for Ethics and Society, The University of Notre Dame Australia, Sydney, Australia; 4 Institute of Palliative Care, Paracelsus Medical University, Salzburg, Austria

**Keywords:** Spiritual distress, Brain tumor, Attitude, Spiritual care, Neuro-oncology

## Abstract

**Purpose:**

People with primary malignant brain tumors experience serious health-related suffering caused by limited prognosis and high symptom burden. Consequently, neuro-oncological healthcare workers can be affected emotionally in a negative way. The aim of this study was to analyze the attitudes and behavior of nurses and physicians when confronted with spiritual distress in these patients.

**Methods:**

Neurospirit-DE is a qualitative vignette–based, multicenter, cross-sectional online survey that was conducted in Bavaria, Germany. Reflexive thematic analysis was used for data analysis.

**Results:**

A total of 143 nurses and physicians working in neurological and neurosurgical wards in 46 hospitals participated in the survey. The participants questioned if the ability to provide spiritual care can be learned or is a natural skill. Spiritual care as a responsibility of the whole team was highlighted, and the staff reflected on the appropriate way of involving spiritual care experts. The main limitations to spiritual care were a lack of time and not viewing spiritual engagement as part of the professional role. Some were able to personally benefit from spiritual conversations with patients, but many participants criticized the perceived emotional burden while expressing the imminent need for specific training and team reflection.

**Conclusions:**

Most neuro-oncological nurses and physicians perceive spiritual care as part of their duty and know how to alleviate the patient’s spiritual distress. Nonetheless, validation of spiritual assessment tools for neuro-oncology and standardized documentation of patients’ distress, shared interprofessional training, and reflection on the professional and personal challenges faced when confronted with spiritual care in neuro-oncology require further improvement and training.

**Supplementary Information:**

The online version contains supplementary material available at 10.1007/s00520-024-08704-y.

## Introduction

Spirituality is recognized by the World Health Organization as one of the fundamental pillars of health and well-being [1]. Frequently, authors argue that spirituality is difficult to define, because of its multidimensional nature [2–4]. According to the European Association for Palliative Care (EAPC), the spiritual field encompasses existential questions, value-based considerations and attitudes, and religious considerations and foundations. “Spirituality is the dynamic dimension of human life that relates to the way persons (individual and community) experience, express and/or seek meaning, purpose and transcendence, and the way they connect to the moment, to self, to others, to nature, to the significant and/or the sacred” [5].

Patients with brain tumors present themselves with a unique combination of symptoms of both neoplastic and neurological disease, including aphasia, neurocognitive deterioration, hemiparesis, seizures, and tumor fatigue [6, 7]. This results in a more complex interaction with the patient and impairs communication and decision making for instance in the presence of aphasia or other neurocognitive deficits [7]. Current research [8–15] on the spiritual needs of neuro-oncological patients and health professionals' attitudes toward spiritual care in this highly specialized field is limited. Spiritual care protocols for patient-centered care are lacking [13, 16], and as brain tumor patients can suffer from rapid neurocognitive deterioration, patients’ spiritual needs are often neglected [8]. It has been demonstrated that brain tumor patients have dynamic spiritual needs throughout the disease course that are not always recognized or addressed by staff because they are hesitant or do not know how [12, 17]. Existential concerns among patients were shown to be more acute at initial diagnosis, reduce during treatment, and may rise again with the end of therapy or tumor progression, mirroring the fluctuating course of the disease [16, 17]. While there are some specific assessments of spiritual care for neuro-oncological patients, the research on the effectiveness of these assessments is still limited.

Throughout the disease trajectory, proactively screening for spiritual needs with simple, validated questions like “Are you at peace?” [18] or psychological screening tools like the functional assessment of chronic illness therapy-spiritual well-being scale (FACIT-Sp) is recommended as a standard procedure in palliative care [19]. The EAPC defines four competencies that ideally constitute spiritual care education as part of interdisciplinary palliative care [4, 20]: (1) reflect on the importance of one’s own spirituality, (2) recognize the role spirituality plays in the life of the patients, understand and respect their needs and choices, (3) address spiritual care needs and document spiritual care provision, and (4) respect personal boundaries.

They further highlight the need for training every health care worker to become a spiritual care generalist with referral of patients to a spiritual care specialist if required [4]. The EAPC also calls for an implementation of shared spiritual care training for all specialties because the multidisciplinary approach to spiritual care seems beneficial [17, 21]. They recommend taking a standardized spiritual history of patients upon hospitalization [20] with the help of tools like the FICA [22] or HOPE [23], which are established and validated, but still need individual adaptation [24].

Although spiritual care services have become recognized as a service within oncology and palliative care, organizational and operational issues are underrecognized yet significant factors in the success of spiritual care programs [25]. This study investigates the reactions of nurses and physicians from neurological and neurosurgical units toward spiritual care for patients with primary malignant brain tumors.

## Methods

### Participants and study design

A qualitative, multicenter, cross-sectional, vignette-based online survey was chosen as the ideal study design to promote a heterogeneous participant sample, high participation rates, and time- and cost-effective distribution [26]. Vignettes have proven to be a valuable research tool in the social and health sciences. Findings of vignette-based studies have the potential to bridge the gap between self-perceived competencies and actual practice, which is essential to further develop spiritual care education and implementation [27]. To ensure broad representation of different approaches to spiritual care, nurses as well as physicians working in neurological and neurosurgical wards were eligible to participate in this study.

### Recruitment

A list of all 184 neurological and neurosurgical departments in Bavaria was compiled by an online search in December 2022, and then, they were subsequently contacted via e-mail or phone from February to April 2023. In total, 20 nursing teams and 21 chairs of departments or head attending physicians and their teams agreed to participate (22.3% response rate) (Supplementary Information 1).

### Data collection

**Fig. 1:**
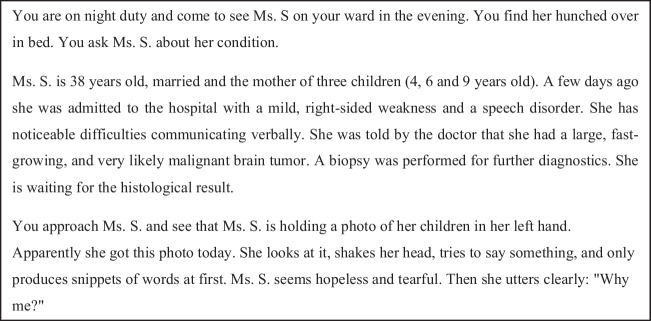
The validated vignette, from Grabenweger et al. (2023) (translated into English, original in German)

**Fig. 2:**
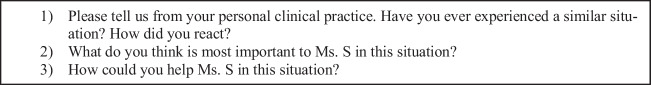
Three open-ended questions asked in the questionnaire (translated into English, original in German)

The online survey was conducted via SoSciSurvey.com. The study was approved by the Regensburg University Institutional Ethics Review Board (vote no. 22–3102-101), and all participants had to read the privacy policy and digitally consent before accessing the questionnaire. The survey contained quantitative questions about participants’ basic demographics, self-reported spirituality and religiosity, and work experience, followed by one vignette (Fig. 1) with three open-ended questions (Fig. 2). To increase the depth and richness of responses, three relatively similar open-ended questions were asked while keeping the questionnaire as short as possible to reduce the risk of attrition. Not all questions were mandatory.

On the first page of the questionnaire was a definition of the key term “spirituality” [5] to clearly state the broad understanding of the concept underlying our study. The original questionnaire can be found in the supplement (Supplementary Information 2).

The pre-tested electronic survey was open from April 16 to May 14, 2023, and easily accessible to participants via link or QR code sent to them by their head attending physicians or chief nursing officers via e-mail (Supplementary Information 3).

### Data analysis

Basic quantitative statistical analysis was performed using IBM SPSS Statistics (29.0.0.0, 241). The analysis of the qualitative data was guided by Braun and Clarke’s (2022) reflexive thematic analysis (RTA) approach [28]. Unlike other forms of thematic analysis, RTA focuses on the organic development of codes, the smallest subunit of shared meaning, and their subsequent iterative and active development into larger themes. In RTA, the subjectivity of the researcher is seen as an advantage rather than a limitation of the method, helping to find meaning and themes in implicit and explicit ways. Nonetheless, the researchers own positioning within the social structure of a Western country and normative expectations within a secular society in Europe inevitably shape data analysis and must be kept in mind while interpreting our findings. The primary coder DV is a 23-year-old female medical student from southern Germany. She does not follow any religion nor identifies as spiritual, but since she grew up in a catholic society, some influence might be present.

Based on this method, our goal was to develop themes that go deeper than the superficial description of participants’ responses and to find meaning beyond the articulated words in an inductive way.

Due to the semantic challenges, a nuanced translation from German into English was required for the codes, themes, and quotations in preparation for this paper. This was challenging and therefore done and accurately documented in a consensus discussion with native speakers who are also well versed in spiritual care (MB, KJ) (Supplementary Information 4). A total of eight themes were developed from the dataset. A graphic overview in the form of a thematic map can be found in the supplements (Supplementary Information 5). This paper reports only the theme that focuses on the reactions of neuro-oncological nurses and physicians to the patient’s spiritual distress to allow for a richer and deeper analysis, following the recommendations of Braun and Clarke [28].

## Results

### Characteristics of participants

A total of 152 individuals commenced the survey, but only the 143 participants who completed the questionnaire up to the first open-ended question about spiritual care were included.

Of the 143 enrolled participants, 66.4% identified themselves as female and 33.6% as male, and their mean age was 41.9 years (range: 23–71 years). Approximately half of them worked as nurses (*n* = 75) and the other half as physicians (*n* = 68), with neurology as the main professional field (57.3%). The years of work experience varied among the participants, reflecting the age range reported. This indicates that the data set provides a wide range of diverse views across different generations and levels of personal experience with patients. A table with more detailed participants’ characteristics is included in the supplements (Supplementary Information 6).

In terms of self-reported spirituality and religiosity, participants predominantly described themselves as somewhat, or a little, religious or spiritual, with a minority reporting that they did not feel spiritual or religious at all (26.6% and 27.3%, respectively). Only eight individuals described themselves as very spiritual and only four as very religious.

In addition, more than 80% of participants (91% of physicians and 76% of nurses) stated they recognized the situation described in the vignette, indicating high external validity of our study.

### Findings of the reflexive thematic analysis

The theme of our reflexive thematic analysis that is reported here deals with the role of the person who works in a health profession and is confronted with spiritual care in this context. We have named it “A Calling and the one called – reacting to distress”. It is only one part out of the eight themes that were generated in total and consists of five subthemes that represent different attitudes. Moreover, a second, smaller theme (“Let me pass, I’m a doctor”) was added to the over-defining central theme as a new subtheme, because its content represents an antithesis to the theme under focus but shares the same fundamental idea. So overall, six interrelated attitudes and reactions, the “sub-themes”, are described below. As the focus of this manuscript is on the attitudes of healthcare workers, we elected only the themes that present these attitudes. The remaining themes report the actions and external practices of healthcare workers (Supplementary Information 5). This allocation into separate conceptual approaches gives space for a richer analysis of the core concept of the theme [28]: different ways in which healthcare workers feel called or avoid spiritual care when directly confronted with the spiritual distress of a neuro-oncological patient.

1. **The human being wearing scrubs—natural or acquired skills** raised the question of whether one can learn spiritual care or if some are gifted with this skill and others are not. It was accompanied by the idea that professional experience could be necessary. Thus, the question was raised whether it is reasonable and even possible for younger colleagues to provide spiritual care. Moreover, other character traits such as “emotional stability” (216, nurse) and an “open and cheerful nature” (41, nurse) of the staff were emphasized as beneficial to spiritual care and aptly summarized by the question of whether empathy can be learned: “Empathy is a trait that’s hard to learn” (64, physician).
2. **Together in the same boat—teamwork** highlighted the importance of the whole team working together. The open exchange of all those who are in contact with the patient seemed to be of great importance to jointly form a supportive network that gave the struggling person the feeling that everyone understands their situation and was there for them. This sensitization of colleagues to create a common awareness seemed appropriate to participants during team meetings and handovers. Only in this way, could everyone pull in the same direction. “It is important to clearly show her, that she won’t be left alone in her misery. […] Several medical specialties are working closely together for her and are trying their best” (151, nurse).
3. One of the major limitations, **the daily business—lack of time** was the hectic daily ward routine and straining workload affecting the provision of spiritual care. “I sat next to the patient and tried to comfort her, but I was constantly called off by my telephone or the nurse buzzer” (50, nurse).
4. **Profession as a burden** addresses the emotional reactions that confronting spiritual care can evoke in providers, both positive and negative. For many participants, spiritual care was an emotional challenge. While some described it as “thought-provoking, stimulating […]” (138, nurse) and could use the opportunity for self-reflection and pondering their own human existence, some were burdened and shielded themselves emotionally. “Always with the thought in the back of my mind, what if it happens to me or my loved ones?” (138, nurse).
  For some, one’s own emotional detachment in everyday life was observed and critically questioned. “Sadly, you get desensitized by the work. You realize that when, instead of empathizing with caring relatives, you are annoyed by them” (62, nurse).
  The staff tried to find ways to protect their inner selves and not to be triggered by care situations, especially in the case of demographic similarities to patients. But at the same time participants were also aware of indifference, habituation to tragic fates, or the danger of appearing “cold” (267, nurse) and thus unintentionally also letting the patient feel this distance. “The older I get, the more these situations get to me, despite my professionalism” (170, nurse) and “Nursing staff should be prepared for these encounters; how do I react in the right way? How can nurses find mental relief?” (267, nurse).
5. **“Not me!”—engaging other professionals as experts** refers to the possibility of including professional groups explicitly trained in spiritual care, such as chaplains, psycho-oncologists, or social workers, and was frequently mentioned as a means of improving spiritual care. However, there seemed to be two distinct approaches here: Firstly, there was the aspect of “being allowed to use” these services after having reached one’s own spiritual care limits or having exhausted the potential for spiritual care: “I definitely always offer a consultation with our psycho-oncologists” (48, nurse). Secondly, some respondents hastily expressed this option, and a personal, existential discussion with the patient was seemingly avoided: “Professional help. In other words: not me!” (262, nurse).
6. **Let me pass, I’m a doctor!** summarizes the various ways in which staff escape from the difficult situation (and thus the need to provide spiritual care). One way this might be done in our scenario would be to place emphasis on the still pending biopsy result. It was implied by some participants that the patient was not entitled to existential anxiety before a final diagnosis is made. In addition, a “professional clinical assessment” (212, physician) of the patient was mentioned in part to rule out hemorrhage as a cause of the speech disorder and then to point out the medical treatment options without giving any space to spiritual fears: “I usually keep the conversation at the clinical level” (212, physician). One respondent even stated that he would leave the room immediately because spiritual care was not his job: “I turned around and left the room: Somebody else needs to take care of this” (262, nurse).

## Discussion

Neuro-oncological staff responded in a variety of ways to spiritual care needs of patients with neuro-oncological disease. The question was raised whether the ability to provide spiritual care is a skill that is learned through years of work experience, or a natural skill obtained by only a few individuals. The importance of seeing spiritual care as a responsibility of the whole team was highlighted, and the participants reflected on the appropriate way and timing of involving spiritual care experts and the time and space needed for spiritual care. Some were able to personally benefit from spiritual conversations and deep engagement with the existential fears of patients, but many participants criticized the emotional burden put on them while expressing the urgent need for specific training. The nurses’ and physicians’ self-observed emotional detachment was justified by the lack of alternatives for spiritual self-care.

The differentiated picture that the participants have of what spiritual care consists of clearly shows an existing understanding of the concept and what is expected of them, even if not always under the term “spirituality”. This includes providing a space for the patients’ distress, acknowledging, and validating the patient’s feelings and seeing the patient as an individual with unique needs. These features have been identified in previous research [12, 29–31]. Nevertheless, some healthcare workers reacted in ways that indicated that they do not consider it their task to respond to the spiritual needs of patients, which has also been previously reported [9, 12]. There is an evident fear of making matters worse and uncertainty of how to engage with patients in acute spiritual distress that make the medical staff hesitate to engage in spiritual conversations [12, 32, 33].

Many EAPC competencies for spiritual care are mirrored in the challenges reported by participants in our study and their proposals for improvement. The EAPC recommends basic spiritual care training for every healthcare worker to become a spiritual care generalist through interdisciplinary shared training for which the participants wish for but so far not been offered. Spiritual care is neither a sole nurses’ practice nor a psychologists’ practice, but rather a human practice and should be understood as everybody’s responsibility [34]. Nonetheless, when everybody is equally responsible for spiritual care, nobody may consider it their explicit duty and spiritual care might remain a neglected area of patient care. Thus, documentation of the provision and assessment for spiritual care is necessary. Because of its emotionally challenging nature, providing spiritual care is not the easiest form of care work, and there is an evident risk of every healthcare worker ignoring it. The European Cancer Organization suggests that one person within the multidisciplinary team be assigned the role of a “key worker” whose responsibility is to coordinate the patient’s psychosocial care and ensure that all other members of the team are aware of the patient’s spiritual needs and address them in a structured way to reduce the aforementioned lack of ignorance [35]. Additionally, evidence-based tools need to be established as part of the palliative patient care routine. Even though the EAPC states the benefits of performing standardized spiritual assessment upon hospitalization using validated questionnaires, a retrospective analysis of spiritual assessment rates among 100 terminally ill patients with glioblastoma at a US academic medical center showed that no patient received any spiritual assessment at more than 50% of their clinical [36]. As cognitive decline is common in patients with brain tumors, the use of formal assessment scales is restricted, and the need for adaptation of tools for the neuro-oncological patient collective is clear [36]. Most scales are not yet validated for brain tumor patients, and only the distress thermometer [37] has been successfully validated as a screening tool for psycho-oncological needs in high-grade glioma patients by Philip et al. [16].

Similar to the recommendations of the EAPC [4], we could also identify self-reflection of the neuro-oncological staff to be of central importance to spiritual care, because establishing a genuine relationship with the patient may also represent an intimate experience with one’s own self to which one must be open. Standardized tools for self-reflection such as the Maslach burnout inventory can be used by healthcare workers to improve this process [38]. A recent study suggests that staff’s individual characteristics and openness to patients are more important than their profession when discussing spiritual issues [39]. As this can be a strain on the healthcare workers [9, 12], setting personal boundaries becomes necessary. There is also the question raised by some participants whether it is unprofessional to be overly burdened with this aspect of one’s chosen profession. The staff is faced with a conflict of responsibilities because other tasks and patients should not be neglected, nor should the patient, who is in need at that moment, be made to feel that he or she is not seen as a priority because of constant external disturbances. This is where the importance of spiritual care becomes clear, and each staff member must find their own way to prioritize what is most important at any one time.

The EAPC explicitly includes the training of health care workers in self-reflection on their own spirituality in their recommendations of education on spiritual care [4]. This can be done through informal team meetings, but also in more structured ways in the form of Balint Groups, Schwartz Rounds, or guided self-assessment and paves the way for nurses and physicians to then be more comfortable with providing spiritual care for patients [4]. As a secondary effect, structured team meetings and frequently nurturing one’s own spiritual well-being have been shown to reduce the risk of burn-out [40, 41]. To our knowledge, there is no data available yet on the implementation of these groups in the neuro-oncological setting. None of the participants mentioned the possibility of joining such groups. Educating healthcare workers on available resources to gain introspection and engage with their own spirituality while also teaching emotional coping strategies appears necessary and a promising way to improve spiritual care.

Our findings highlight the need for spiritual care to be treated as an interdisciplinary team effort [17, 21]. We found that, by building strong interpersonal relationships with their patients and seeing them as an individual, healthcare professionals can alleviate spiritual distress and improve patient care, as seen in other studies [39, 42]. However, most authors refer to spiritual care as a nursing practice, and most data is based on nurses’ experiences [32, 33, 43, 44]. Since we explored both nurses’ and physicians’ attitudes on spiritual care, new data to the interdisciplinary approach is added. An Australian study also found that all members of the healthcare team feel responsible for addressing spiritual distress [45], especially when they had received specific training beforehand [46]. However, there seems to be a lack of open sharing within the healthcare team about the spiritual distress of patients, maybe also due to the absence of standardized documentation [36]. Spiritual care can impose an emotional challenge on the individual, and while the team approach can provide stability and a source of strength for every team member, the risk of individuals hiding behind the team, avoiding personal confrontation with existential questions, and not feeling like the best equipped team member for alleviating patients’ spiritual distress needs to be considered. When spiritual care is deemed as a team responsibility that is frequently discussed during handovers, the communication on which team member is providing spiritual care must be open and thorough to avoid this confusion.

In methodological terms, the information power of this study seems appropriate, because the topic studied is specific, as is our participating group of medical professionals [47]. The large participant sample must be considered one of the main strengths of this qualitative analysis. Even though there is likely to be some level of selection bias, our participant group appears to represent a spiritually diverse subset of the German population [48].

However, there are some limitations regarding cultural diversity and using a remote online study for data collection. It must be noted that the survey is based within the context of a secular society in a Western country, and thus, the transferability of the findings for other cultures must at least be questioned. Nevertheless, the vignette represents a rather typified situation with a young mother and characteristics such as ethnicity or accent are purposefully left to the imagination of the respondents. When using an online survey, participants are very flexible regarding time and space, but disadvantages include the need for access to the Internet and a private device and time to fill in the survey, literacy, and the risk of trolling or unfaithful participation of people outside the desired study population [26]. Hence, data items need to be critically appraised.

## Conclusion

We report on various reactions of the neuro-oncological staff when confronted with spiritual distress of a patient and were able to identify several areas in need of improvement to further secure the role of spiritual care for patients with brain tumors. Many participants stated that they do not feel well-prepared for this task. Even though the use of formal spiritual assessment is recommended, along with ensuring that every health care professional can perform a basic spiritual assessment and be aware of techniques to reduce spiritual distress, rates of reported spiritual care are low, not routinely performed, and most tools are not validated for brain tumor patients. Implementing spiritual care training as a regular part of professional education could form the basis for enabling healthcare workers to self-reflect and provide spiritual care, and therefore, it seems reasonable to start educating students from early on. Furthermore, shared training of physicians and nurses should be implemented alongside regular interdisciplinary team discussions on spiritually distressed patients to support them together as one healthcare team in everyday working life. To increase security regarding spiritual care within one’s role as a healthcare worker and reduce the risk of ignorance towards the patients’ distress and emotional burn-out of the staff, self-reflection on spirituality is helpful. This can be taught in structured groups or done individually with the aim of establishing the spiritual engagement with patients as an essential part of palliative care, benefitting both the staff and the patients alike.

### Supplementary Information

Below is the link to the electronic supplementary material.

Supplementary file1 (PDF 74 KB)

Supplementary file2 (PDF 921 KB)

Supplementary file3 (PDF 150 KB)

Supplementary file4 (PDF 231 KB)

Supplementary file5 (PDF 85 KB)

Supplementary file6 (PDF 158 KB)

## Data Availability

The original dataset generated by the survey is not available online but can be obtained from the authors upon reasonable request.
