# Supplementary material for: “Not me!” a qualitative, vignette-based study of nurses’ and physicians’ reactions to spiritual distress on neuro-oncological units
Source: Support Care Cancer. 2024 Jul 10;32(8):499. doi: 10.1007/s00520-024-08704-y (PMC11236889; doi:10.1007/s00520-024-08704-y)

Supplementary Information (1)

Flow Chart demonstrating the route of participant recruitment from December 2022 until April 2023

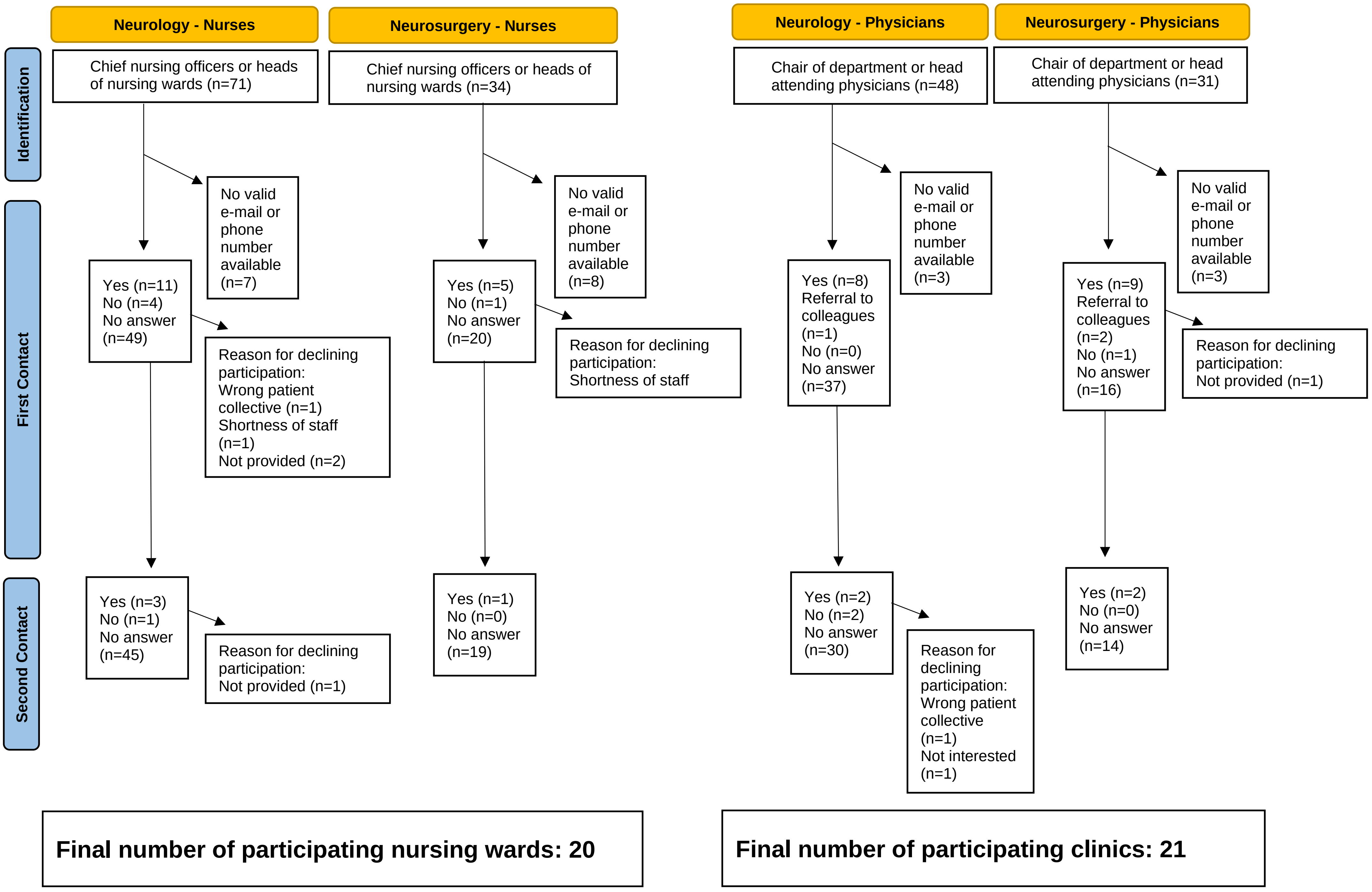

Supplement: Supplementary file 1 — Supplementary file1 (PDF 74 KB) [file 520_2024_8704_MOESM1_ESM.pdf]
