# Supplementary material for: “Not me!” a qualitative, vignette-based study of nurses’ and physicians’ reactions to spiritual distress on neuro-oncological units
Source: Support Care Cancer. 2024 Jul 10;32(8):499. doi: 10.1007/s00520-024-08704-y (PMC11236889; doi:10.1007/s00520-024-08704-y)
Supplement: Supplementary file 2 — Supplementary file2 (PDF 921 KB) [file 520_2024_8704_MOESM2_ESM.pdf]

Original online questionnaire in German, downloaded from SoSciSurvey.com on the 15th of May 2023

Homepage

SC16

Dear Participant,

Thank you for your interest in participating in our online survey! First of all, some information about the study for you as study participants:

Study Objective:

Patients with malignant brain tumors have a very limited prognosis, and at the same time they and their relatives are extraordinarily burdened by the accompanying neurocognitive impairments, speech disorders or pareses. It has already been shown that there is an increased need for psycho-oncological, palliative and spiritual support because of this. Despite the great importance for this particularly burdened patient group, the data on the topic of spiritual care in neuro-oncological patients is still sparse. The aim of our study is to assess the different attitudes of nursing and medical staff towards spiritual care and to derive adequate measures with regard to the spiritual needs of people with malignant brain tumors.

Underlying Spirituality:

"Spirituality is the dynamic dimension of human life that refers to how persons (individually and in community) experience, express, and/or seek meaning, significance, and transcendence, and how they relate to the moment, self, other/m, nature, signifier, and/or sacred."

(European Association for Palliative Care - EAPC. Task force. What is spiritual care? [Translation: Kammerer, Roser & Frick]).

Participation Requirements:

- Employee* in the nursing and medical area
- Field of activity on a neurosurgical or neurological ward in Bavaria

Time required:

Approximately 15 minutes are recommended for completing the questionnaire.

You will find information on ethics and data protection on the next page. Please just click on!

SC17

Declaration of consent

This survey is conducted as part of a medical dissertation at the University Hospital Regensburg. The collected, sensitive data will be handled in accordance with the DSGVO, although only anonymous data will be collected. In principle, data may be stored without restriction according to Art. 89 (1) of the DSGVO.

You have the right to be informed by the person responsible for the study about the data collected as well as the right to correction, deletion, restriction of the processing of your data as well as the right to object to the processing and the right to data portability. If you would like to exercise your rights afterwards, please note your contact address in the "What else I wanted to say" field at the end of the questionnaire.

Before you can start the questionnaire, you will receive detailed information about your rights in the course of this survey and will be asked for your consent.

You have the following **personal rights** as part of this survey:

- Participation in this study is **voluntary** and you may stop the study at any point.
- Your participation is absolutely **anonymous**. This means that your answers cannot be traced back to you. Your personal data set is not identifiable after completion of the survey.
- Your data will be used expressly for **scientific purposes.** It may happen that your data are used in the context of other research projects of the study responsibles. be processed further.
- This research project does not follow any commercial interests. Your data will be treated **strictly confidential.**

If you have any questions about this data collection, please feel free to contact:

Elisabeth Bumes, MD

Clinic and Polyclinic for Neurology at the University Hospital Regensburg

Franz-Josef-Strauß-Allee 11

93053 Regensburg, Germany

E-mail: elisabeth.bumes@ukr.de

SC19

Use of the collected data:

The data of this study are collected and used for the purpose of research and especially for a scientific publication. Access to the raw data is exclusively granted to members of the neuro-oncology study team at the University Hospital Regensburg. A transfer of the data to external parties or to recipients in third countries is not intended. The data will be deleted after a
deleted after a retention period of 10 years. The publication of results takes place in anonymized form, i.e. without the data being able to be assigned to a specific person. Should you indicate your interest in the study results, your contact data will be collected separately from your answers to the questionnaire and used only for this purpose.

Privacy:

The legal basis for the data processing is your consent pursuant to Art. 6 (1) a and Art. 9 (2) a DSGVO, which you give us together with the answer to the questionnaire. The
The responsible party for data processing is: University Hospital Regensburg, Franz-Josef-Strauß-Allee 11, 9303 Regensburg.

The data will be treated confidentially at all times. They are only used in pseudonymized or evaluated in anonymized form and not passed on to third parties. Personal data such as
Gender and occupational group do not allow any clear conclusion about your person. The data collected in addition to this does not allow any conclusion to be drawn about your person either. However, identification is not intended even if, in exceptional cases, your identity could be inferred from the data.

Can I revoke my consent?

Your participation in this examination is voluntary. You can revoke your respective consent in writing or verbally at any time without giving reasons and without incurring any disadvantage. If you revoke your consent, no further data will be collected. However, the data processing that took place until the revocation remains lawful. In the event of revocation, you can also request the deletion
of your data. However, deletion is only possible insofar as an assignment of the questionnaire to your person can be made.

What other rights do I have with regard to data protection?

You have the right to request information from the person responsible about the personal data stored about you (including the provision of a copy of the data free of charge). Likewise, you may request the correction of inaccurate data and, if necessary, a transfer of the data you have provided to the
provided data and request the restriction of their processing. As a rule, please contact the contact person for the study named below.

If you have any questions regarding data protection, you are also welcome to contact the UKR data protection officer:

Dr. Wolfgang Börner, Franz Josef-Strauß Allee 11, 93035 Regensburg, dsb@ukr.de, Tel: 0941 944-0

Furthermore, there is a right of appeal to any supervisory authority. Responsible for the UKR is:

The Bavarian State Commissioner for Data Protection (poststelle@datenschutz-bayern.de, Postfach 22 12 19, 80502 Munich, Tel: 089 212672-0)

Contact for questions about the study:

The study is being conducted by Elisabeth Bumes, MD. If you have any questions about this study, please contact:

Elisabeth Bumes, MD

Clinic and Polyclinic for Neurology at the University Hospital Regensburg

Franz-Josef-Strauß-Allee 11 93053 Regensburg, Germany

E-mail: elisabeth.bumes@ukr.de

Phone: 0941-944-18751

Opt Out

SC18

1. I hereby confirm that I have read and understood the consent form and I agree with the above mentioned conditions of participation:

◯ Yes

◯ No (do not participate in this study)

1 active filter(s)

Filter SC18/F1
If one of the following response option(s) is selected: **2**
Then after clicking "Next" display the text **SC13** and finish the interview

---

Occupation

SC20

2. Which professional group do you belong to?
Please select an answer option:

O Physician
O Nurse

SC24

3. In which area do you work?
Please select an answer option.

O Predominantly in Neurology
O Predominantly in Neurosurgery
O Both

SC07

4. Please enter your age in whole years!

Only numbers may be entered in this field. Please enter your answer here:

SC08

5. Please specify your gender!

Please select only one of the following answers

O Female
O Male
O Diverse
O No answer

SC09

6. Please specify your work experience

Please select only one of the following answers:

- 0-5 years
- 6-10 years
- 11-15 years
- 16-20 years
- 21-25 years
- 26-30 years
- 31-35 years
- 36-40 years
- 41-45 years
- 46-50 years
- No specification

SC10

7. Regardless of your attitude toward certain beliefs or certain religions, how would you describe yourself?

Please select only one of the following answers

O Very spiritual
O Somewhat spiritual
O A little spiritual
O Not spiritual
O Choose not to say

SC11

8. Regardless of whether you belong to a religious community or attend a religious service, how would you describe yourself?

Please select only one of the following answers:

O Very religious
O Somewhat religious
O A little religious
O Not religious
O Choose not to say

SC01

Please read the following situation description:

You are on night duty and come to Mrs. S. on your ward in the evening. You find her sitting curled up in bed. Now you ask Ms. S about her condition.

Mrs. S. is 38 years old, married and mother of three children (4, 6 and 9 years). A few days ago, she presented for inpatient admission with mild right-sided weakness and a speech disorder. She has noticeable difficulty communicating verbally. She was told by a doctor that she had a large, fast-growing and very likely malignant brain tumor. A biopsy was performed for further diagnosis. The histological result is currently pending.

They approach Ms. S. and see that Ms. S. is holding a photo of her children in her left hand. Apparently she received this photo today. She looks at it, shakes her head, tries to say something and for the time being only produces word fragments. Mrs. S. seems hopeless and tearful. Then she clearly states, "Why me?"

SC02

9. Do you know such a situation from your everyday professional life?
Please choose one of the following answers

- ◯ Yes
- ◯ No
- ◯ No data

1 active filter(s)

Filter SC02/F1
If one of the following response option(s) is selected: **1**
Then show page(s) **Vignette 2** of the questionnaire (otherwise hide).

SC22

To answer the following questions, the situation is described again here:

You are on night duty and come to Mrs. S. on your ward in the evening. You find her sitting curled up in bed. Now you ask Ms. S about her condition.

Mrs. S. is 38 years old, married and mother of three children (4, 6 and 9 years). A few days ago, she presented for inpatient admission with mild right-sided weakness and a speech disorder. She has noticeable difficulty communicating verbally. She was told by a doctor that she had a large, fast-growing and very likely malignant brain tumor. A biopsy was performed for further diagnosis. The histological result is currently pending.

They approach Ms. S. and see that Ms. S. is holding a photo of her children in her left hand. Apparently she received this photo today. She looks at it, shakes her head, tries to say something and for the time being only produces word fragments. Mrs. S. seems hopeless and tearful. Then she clearly states, "Why me?"

SC03

10. Please tell us from your professional practice! Have you ever been in a similar situation? How did you react?

Please enter your answer here:

SC04

To answer the following questions, the situation is described again here:

You are on night duty and come to Mrs. S. on your ward in the evening. You find her sitting curled up in bed. Now you ask Ms. S about her condition.

Mrs. S. is 38 years old, married and mother of three children (4, 6 and 9 years). A few days ago, she presented for inpatient admission with mild right-sided weakness and a speech disorder. She has noticeable difficulty communicating verbally. She was told by a doctor that she had a large, fast-growing and very likely malignant brain tumor. A biopsy was performed for further diagnosis. The histological result is currently pending.

They approach Ms. S. and see that Ms. S. is holding a photo of her children in her left hand. Apparently she received this photo today. She looks at it, shakes her head, tries to say something and for the time being only produces word fragments. Mrs. S. seems hopeless and tearful. Then she clearly states, "Why me?"

SC05

11. What do you think is the most important thing for Mrs. S. in this situation?

Please enter your answer here:

To answer the following questions, the situation is described again here:

SC23

You are on night duty and come to Mrs. S. on your ward in the evening. You find her sitting curled up in bed. Now you ask Ms. S about her condition.

Mrs. S. is 38 years old, married and mother of three children (4, 6 and 9 years). A few days ago, she presented for inpatient admission with mild right-sided weakness and a speech disorder. She has noticeable difficulty communicating verbally. She was told by a doctor that she had a large, fast-growing and very likely malignant brain tumor. A biopsy was performed for further diagnosis. The histological result is currently pending.

They approach Ms. S. and see that Ms. S. is holding a photo of her children in her left hand. Apparently she received this photo today. She looks at it, shakes her head, tries to say something and for the time being only produces word fragments. Mrs. S. seems hopeless and tearful. Then she clearly states, "Why me?"

SC06

12. How do you think you can help Mrs. S. in this situation?

Please enter your answer here:

Study results

SC12

13. Are you interested in the study results?

Please select only one of the following answers:

- ◯ No
- ◯ Yes

1 active filter(s)

Filter SC12/F1
If one of the following response option(s) is selected: **2**
Then show question/text **SC13** later in questionnaire (otherwise hide)

SC13

Thank you for your interest in our work!

Please contact the researchers at the following email address to receive the study results upon completion:

daniela.voelz@stud.uni-regensburg.de

Comments

SC14

14. What else you wanted to say:
Please enter your answer here:

SC15

Dear Participants,

Thank you for your participation! You are helping us to learn more about spirituality in the care and treatment of people with malignant brain tumors. The case vignette presented in the survey was theoretically constructed and does not reflect reality.

You have now reached the end of the questionnaire!

If you still have questions about this survey and/or are interested in the results of this study, please contact daniela.voelz@stud.uni-regensburg.de.

Please click on the "Continue" button. This will close the questionnaire and your data will be saved.

Thank you for your participation!

You may now close this website!

Would you like to participate in interesting and exciting online surveys in the future?

We would be very pleased if you register your e-mail address for the SoSci Panel and thus support scientific research projects.

Email: Join the panel

Participation in the SoSci Panel is voluntary, non-binding and can be revoked at any time. SoSci Panel does not store your email address without your consent, does not send you advertisements, and does not share your email address with third parties.

Of course, you can also close the browser window without participating in the SoSci Panel.

Elisabeth Bumes - 2023

Original online questionnaire in German, downloaded from SoSciSurvey.com on the 15th of May 2023

Supplementary Information (2) in English
