# Supplementary material for: “Not me!” a qualitative, vignette-based study of nurses’ and physicians’ reactions to spiritual distress on neuro-oncological units
Source: Support Care Cancer. 2024 Jul 10;32(8):499. doi: 10.1007/s00520-024-08704-y (PMC11236889; doi:10.1007/s00520-024-08704-y)
Supplement: Supplementary file 3 — Supplementary file3 (PDF 150 KB) [file 520_2024_8704_MOESM3_ESM.pdf]

Supplementary Information (3)

Letter to participate, sent out via E-Mail in April of 2023 in German, English translation

Betreff: **Neurospirit – Link zur Onlineumfrage zu Spiritual Care bei Hirntumorpatient*innen**

Sehr geehrter Herr/Frau […] ,

Wir möchten uns nochmals recht herzlich bedanken, dass Sie und Ihr Ärzteteam/Pflegeteam der Neurologie / Neurochirurgie sich bereit erklärt haben, an unserer Umfrage zur Spiritual Care bei Patienten mit primären malignen Hirntumoren teilzunehmen.

Hier senden wir Ihnen wie angekündigt den Link zu dem Onlinefragebogen zu:

https://www.soscisurvey.de/neurospiritsurvey/

Bitte leiten Sie diesen an alle Ärzt*innen/Pflegekräfte in Ihrem Bereich weiter.

Außerdem finden Sie im Anhang dieser E-Mail einen QR-Code, der ebenfalls zu dem Fragebogen führt. Somit können Interessierte auch bequem per Smartphone teilnehmen.

Die Umfrage ist **vom 17. April bis 14. Mai 2023** zur Teilnahme geöffnet und nimmt 10-15 Minuten Zeit in Anspruch. Für Rücksprachen stehen wir weiterhin jederzeit per E-Mail oder telefonisch unter +49 (0) 941/ 944-18751 zur Verfügung.

Für Interessierte gibt es außerdem die Möglichkeit, sich im Fragebogen in eine Liste eintragen zu lassen. Dann lassen wir Ihnen nach Auswertung der Daten die Ergebnisse zukommen.

Wir freuen uns über Ihre zahlreiche Teilnahme!

Beste Grüße

Dr. med. Elisabeth Bumes

Oberärztin Neurologie
Universitätsklinikum Regensburg
Klinik und Poliklinik für Neurologie – NeuroOnkologie
Franz-Joseph-Strauß-Allee 11
93053 Regensburg
Deutschland (GER)
Fon: +49 (0) 941/ 944-18751
Fax: +49 (0) 941/ 944-38756
E-Mail: elisabeth.bumes@ukr.de
Homepage: www.ukr.de/zht

Daniela Völz

Doktorandin Neurologie
Universitätsklinikum Regensburg
Franz-Joseph-Strauß-Allee 11
93053 Regensburg
Deutschland (GER)
E-Mail: Daniela.Voelz@stud.uni-regensburg.de

Subject: **Neurospirit - Link to the online survey on spiritual care for brain tumor patients**

Dear Mr./Mrs. [...] ,

Once again, we would like to thank you and your team of doctors/nursing staff in neurology/neurosurgery for agreeing to take part in our survey on spiritual care for patients with primary malignant brain tumors.

As previously announced, we are sending you the link to the online questionnaire here:

https://www.soscisurvey.de/neurospiritsurvey/

Please forward this to all doctors/nurses on your ward.

You will also find a QR code attached to this e-mail, which also leads to the questionnaire. This means that interested participants can also take part conveniently via smartphone.

The survey is open for participation from **April 17 to May 14, 2023** and takes 10-15 minutes to complete. We are still available for consultation at any time by e-mail or by telephone at +49 (0) 941/ 944-18751.

Interested participants also have the option of registering on a list in the questionnaire. We will then send you the results after evaluating the data.

We look forward to your participation!

Best regards

Dr. med. Elisabeth Bumes

Senior Physician Neurology
University Hospital Regensburg
Clinic and Polyclinic for Neurology – NeuroOncology
Franz-Joseph-Strauß-Allee 11
93053 Regensburg
Deutschland (GER)
Fon: +49 (0) 941/ 944-18751
Fax: +49 (0) 941/ 944-38756
E-Mail: elisabeth.bumes@ukr.de
Homepage: www.ukr.de/zht

Daniela Völz

Doctoral Candidate Neurology
University Hospital Regensburg
Clinic and Polyclinic for Neurology - NeuroOncology
Franz-Joseph-Strauß-Allee 11
93053 Regensburg
Deutschland (GER)
daniela.voelz@stud.uni-regensburg.de
