# Supplementary material for: “Not me!” a qualitative, vignette-based study of nurses’ and physicians’ reactions to spiritual distress on neuro-oncological units
Source: Support Care Cancer. 2024 Jul 10;32(8):499. doi: 10.1007/s00520-024-08704-y (PMC11236889; doi:10.1007/s00520-024-08704-y)
Supplement: Supplementary file 4 — Supplementary file4 (PDF 231 KB) [file 520_2024_8704_MOESM4_ESM.pdf]

Supplementary Information (4)

**Translation of the Original Quotes into English**

With the help of MB and KJ, PP, DV and RG

| Original Quote | Translation | Comments |
|---|---|---|
| *„emotionale Stabilität" (216)* | "emotional stability" | IS Emotionally balanced OR HAS emotional stability |
| *„offenes, fröhliches Grundnaturell" (41)* | "generally open and cheerful nature" | Can drop 'generally' |
| *„Empathie ist eine Eigenschaft, die schwer zu lernen ist." (64)* | "Empathy is a trait that's hard to learn." | Eigenschaft = character, trait<br>Hard = difficult |
| *„[Wichtig ist,] ihr deutlich [zu] zeigen, dass sie in ihrer Not nicht alleine gelassen wird. […] Mehrere med[izinische] Fachrichtungen arbeiten für sie eng zusammen und geben ihr Bestes." (151)* | „It is important to clearly show her, that she won't be left alone in her misery. […] Several medical specialties are working closely together for her and are trying their best." | Medical specialities = areas<br>In her Misery = in her time of need |
| *„Ich habe mich zur Patientin gesetzt und versucht sie zu trösten, leider wurde ich durch Telefon und Patientenruf ständig weggerufen." (50)* | "I sat next to the patient and tried to comfort her, but I was constantly called off by my telephone or the nurse buzzer." | Off = away |
| *„nachdenklich, stimulierend […]" (138)* | "thought-provoking, stimulating […]" | |
| *„Auch immer mit dem Hintergedanken, was ist [,] wenn es mich oder meine Angehörigen erwischt?" (138)* | "Always with the thought in the back of my mind, what if it happens to me or my loved ones?" | |
| *„Man stumpft im Alltag leider etwas ab. Das merkt man ganz oft daran, wenn man vielleicht von den besorgten Angehörigen genervt ist, statt sich in deren Situation hineinzuversetzen." (62)* | "Sadly, you get desensitized during the daily worklife. You often realize that when you are annoyed by caring relatives instead of empathizing with their situation." | Sadly, you get desensitized by the work. You realize that when, instead of empathizing with caring relatives, you are annoyed by them. |
| *„eiskalt" (267)* | "ice-cold" | cold |
| *„[Umso] älter ich werde, gehen solche Situationen mir sehr nahe, trotz Professionalität[.]" (170)* | "The older I get, the more these situations get to me, despite my professionalism." | |
| *„Die Pflege sollte auf solche Begegnungen vorbereitet werden, Wie reagiere ich richtig? […] Wie entlastet man die Pflege psychisch?" (267)* | „Nurses should be prepared for these encounters; how do I react in the right way? […] How can nurses be mentally relieved?" | Psychische Entlastung = mental Relief, psychological relief ?<br>Nursing staff should be prepared for these encounters; how do I react in the right way? How can nurses find mental relief |
| *„In jedem Fall biete ich ein Gespräch mit unsere[r] Psychoonkologie an." (48)* | "I definitely always offer a consultation with our psychooncologists." | |
| *„Professionelle Hilfe. In anderen Worten [:] nicht ich!" (262)* | "Professional help. In other words: not me!" | |
| *„Ich haben dann [das] Gespräch meistens auf die fachliche Ebene verlagert[.]" (212)* | "I usually take the conversation to the clinical level." | I usually keep the conversation at the clinical level |
| *„fachlich-klinische Einschätzung" (212)* | "clinical assessment" | Professional clinical assessment |
| *„Umgedreht und rausgegangen [,] soll sich jemand anderes drum kümmern." (262)* | "I turned around and left the room, somebody else should take care of this." | I turned around and left the room: Somebody else needs to take care of this |

| | | |
|---|---|---|
| *„Oft helfen große [W]orte nicht. Aber es hilft auch Berührung und einfach zuhören.“ (200)* | “Often, great words do not help. But it helps to touch them and to just listen.” | Often, words don’t help. But touching and listening helps. |
| *„[I]ch setze mich erstmal zu den Patienten, je nachdem, welcher Patient, näher oder nicht so nah. […] Frauen kann man meist in den Arm nehmen und so da sein……zeigen, du bist nicht alleine [.]“ (252)* | “Firstly, I take a seat next to the patient, depending on the patient sometimes a little closer or not as close. [...] Women usually accept a hug and to be there for them like this … showing them that they’re not alone.” | Firstly, I sit next to the patient. How close depends on the patient. In most cases, you can hug women, just being there for them, showing them they’re not alone. |
| *„Tee kann auch ab und an Wunder wirken [.].“ (191)* | “Tea works wonders from time to time.” | |
| *„[Ich stelle eine] Gegenfrage zu Schmerzen, körperlichem Wohlbefinden, ob man ihr was [G]utes tun könne.“ (56)* | “I ask a counterquestion about pain, physical well-being, or if I can do anything good for her.” | I ask a question about pain, physical wellbeing, or if I can do anything for her. |
| *„medikamentöse Anxiolyse und Schlafinduktion (1 mg Tavor) […] kurze Krisenintervention“ (212)* | „medical anxiolysis and sleep induction (1mg Lorazepam) […] short crisis intervention” | Anxiolytics and hypnotics (1mg lorazepam), acute crisis intervention. |
| *„Kontakt mit Kindern […], da sie der Pat[ientin]meiner Meinung nach am meisten Kraft geben und Motivation weiterzumachen!“ (121)* | „Contact with the children, because in my opinion they give the patient the most strength and motivation to keep going! “ | |
| *„Dass sie weiß, dass nach ihrem Tod gut für ihre Kinder gesorgt ist und dass sie sich kleine Erinnerungen mit ihrer Familie schafft, solange es noch geht. […] Qualitytime mit der Familie ermöglichen [.]“ (268)* | „That she knows that her children will be taken good care of after her passing and that she creates small memories with her family while it’s still possible. […] Enable quality time with the family. | That she knows that her children will be taken care of after her passing and that she can create little memories with her family while it’s still possible. Enable quality time with the family. |
| *„Oft ist es so, dass diese Pat[ienten] zwar sagen, dass sie wen zum Reden haben, aber wenn man nachhakt, merkt man ziemlich schnell, dass sie ihren Angehörigen nicht zur Last fallen wollen […].“ (62)* | „Often, patients say they have somebody to talk to, but when you inquire further, you realize pretty fast that they do not want to be a burden to their loved ones[…].” | Next of kin / loved ones / family / relatives<br>Often, patients say they have somebody to talk to, but when you inquire further, you realise pretty quickly that they don’t want to be a burden on their loved ones |
| *„Ich spreche darüber, dass es leider nicht um Gerechtigkeit geht.“ (188)* | „I talk about the sentiment that, unfortunately, it’s not a question of justice.” | Fact / sentiment<br>Not about / not a question of justice<br>I suggest that, unfortunately, it’s not about justice |
| *„Ich lenke von dieser Frage ab, in dem ich […] auf das Foto anspreche. […] [Das Wichtigste ist] [j]emand, der sich ihrer annimmt, ernst nimmt und sie aus der Gedankenspirale rausholt.“ (124)* | „I divert from this question by […] addressing the photograph. […] The most important thing is that somebody takes care of her, takes her seriously, and gets her out of the thought spiral.” | I create a diversion by focusing on the photograph. The most important thing is that somebody takes care of her, takes her seriously, and stops her spiraling thoughts. |
| *„Diese Frage kann man nicht zufriedenstellend beantworten.“ (26)* | „One cannot give a satisfying answer to this question.” | Provide / give<br>You can’t give a satisfying answer to this question. |
| *„die wichtigste Frage“(191)* | „ the most important question” | To question = hinterfragen<br>To ask = fragen<br>Important/ central / main<br>Have/ ask<br>The main question |
| *„Sie soll nicht […] nach dem „Warum“ fragen, sondern ihr Leben möglichst bewusst und freudig erleben[.]“ (15)* | „She should not ask ‘why’ but rather just live her life as consciously and joyfully as possible.” | Consciously = mindfully |
| *„[Das Wichtigste ist das] Einlassen auf die Sprachstörung mit Aufbau* | „The most important thing is to engage with the aphasia and create | Create/establish<br>The main thing = the most important thing |

| | | |
|---|---|---|
| *der Kommunikation, verbal, schriftlich, Codes[.]" (226)* | verbal and written communication or codes." | |
| *„[Ich bin] sitzen geblieben und habe versucht zu hören, was die Patientin versuchte zu sagen. Es kam zu keinem richtigen Gespräch, die Patientin beruhigte sich aber." (86)* | „I remained seated and tried to listen to what the patient was trying to say. No real conversation developed but the patient did calm down." | |
| *„Ich würde versuchen, mir Zeit für die Patientin zu nehmen […] mich zu ihr setzen und zuhören/warten, ob sie noch weitere klar verständliche Äußerungen von sich gibt. […] [Ich würde] zuhören und gemeinsam mit ihr versuchen, eine Antwort auf ihre Frage zu finden [.]" (152)* | „I would try to take time for the patient […] sitting with her and listening/waiting if she utters any more words thar are clearly comprehensible. […] I would listen and try to find an answer to her question together with her." | Äußerung = statement, remark, word, announcement, phrase<br>I would try to take time for the patient, sitting with her and seeing if she says anything that is clearly comprehensible. I would listen and try to find an answer to her question with her. |
| *„[Ihr zeigen] das[s] die Probleme und Sorgen wahrgenommen werden, das[s] die Pat[ientin] mit der Erkrankung nicht alleine ist und das hier vor Ort geholfen wird, den Funken Hoffnung erhalten [.]" (108)* | „Showing to her, that the problems and worries are noticed, that the patient is not alone with her disease and that she will receive help here, maintain a spark of hope. " | Wahrnehmen = perceive, notice, see<br>Showing her that the problems and worries are recognised, that she is not alone in her illness and she will receive help here – maintain a spark of hope |
| *„[Das Wichtigste ist] Klarheit und kein „Schönreden", jedoch dies empathisch und mit Blick auf die positiven Aspekte, zudem ausreichend Zeit [.]" (88)* | „The most important thing is clarity and no "glossing over", but in an empathetic manner and with an eye on the positive aspects, moreover with sufficient time. " | An eye on/the focus on<br>The main thing is open communication and no glossing over the facts, but with an empathetic manner and focusing on the positive aspects, taking sufficient time |
| *„keine Nummer" (43)* | „not a number" | |
| *„Es ist schwierig in solchen Fällen eine pauschale Lösung zu finden, es ist zu empfehlen auf die individuelle Persona des Patienten einzugehen, und diesen in seiner psychosozialen Verarbeitungsstrategie zu unterstützen." (159)* | "It is difficult to find a one-size-fits-all solution in such cases, it is recommended to respond to the individual persona of the patient, and to support them in their psychosocial processing strategy." | It's difficult to find a one-size-fits-all solution in such cases, you should respond to the individual, and support their psychosocial coping strategy. |
| *„Ich setzte mich neben Frau S. & habe wenig gesprochen. […] Zuhören, Mut machen, gemeinsames schweigen." (102)* | „I sat down next to Ms. S. & did little talking. […] Listening, encouraging, being silent together." | I sat down next to Ms S and didn't talk much. |
| *„Ich kann ihr nicht helfen. Ich kann nur für sie da sein." (41)* | „I can't help her. I can just be there for her." | I can't help her. I can only be there for her |
| *„[E]infach gar nichts sagen und neben ihr sitzen, einfach „da" sein [,] weil manchmal Worte keinen Sinn ergeben." (270)* | „Not saying anything at all and sitting next to her, just being 'there', because sometimes words do not make sense." | |
| *„[Ich würde der Patientin] den Raum geben sich zu äußern, durch Worte, Weinen, Laute …. da bleiben." (245)* | „I would provide the patient with the space to express herself through words, tears, sounds …. Staying there with her." | I would give the patient space to express herself through words etc |
| *„nicht selbst einbrechen" (252)* | „to collapse themselves" | To collapse |
| *„[Ich würde] der Patientin zeigen, dass man versteht, dass die Situation nicht fair ist, [und] eigene Emotionen dazu zeigen." (129)* | „I would signal the patient that you understand the situation is not fair and show your own emotions about it. " | I would indicate to the patient that you understand that the situation is not fair and show my own emotions |

| | | |
|---|---|---|
| *„Ich habe mit der Patientin über die Prognose der Erkrankung gesprochen und auf die Möglichkeit der Therapie hingewiesen. [Außerdem das] Angebot weiterer Hilfestellungen ([...], ausführl[iches], aufklärendes Gespräch)[.]" (177)* | „I talked about the prognosis of the disease and the possibility of therapy. Also the option for further support ([…] an extensive, informative conversation). " | Moreover = also or in addition |
| *„[E]her den weiteren Verlauf oder bisherigen Verlauf verständlich und menschlich zusammenfassen und das [P]ositive hervorheben [...] mit dem Ziel das „Unfassbare" in kleineren Teilen/Schritten „erfassbar" zu machen [.]" (108)* | "Rather summarize the further process or process thus far in an understandable and human way and emphasize the positive [...] with the aim to make the "incomprehensible" "comprehensible" in smaller parts/steps." | Course / trajectory /process<br>Rather summarize the further course and the course so far in an understandable and human way and emphasize the positive with the aim of making the incomprehensible comprehensible step by step. |
| *„gemeinsam eine Strategie entwickeln" (7)* | „Developing a strategy together" | |
| *„Den Sorgen der Patientin zuhören. G[egenebenfalls] realistische Vorschläge zur Lösung der Sorgen vorschlagen (oft betreffen die Sorgen nicht die Angst vor dem Tod, sondern die Frage was aus den Kindern passiert..)[.]" (221)* | „Listening to the patients concerns. Maybe suggest realistic solutions to resolve the worries (often these worries aren't about the fear of dying but rather about what will happen to the children..). | |
| *„Nicht immer muss oder kann eine Lösung präsentiert werden [.]" (43)* | „One can and must not always present a solution." | You can but don't always have to present a solution |

*Translation of the original German quotes into English with the help and comments of MB and KJ*
