# Supplementary material for: “Not me!” a qualitative, vignette-based study of nurses’ and physicians’ reactions to spiritual distress on neuro-oncological units
Source: Support Care Cancer. 2024 Jul 10;32(8):499. doi: 10.1007/s00520-024-08704-y (PMC11236889; doi:10.1007/s00520-024-08704-y)

Supplementary Information (5)

Thematic map of the Reflexive Thematic Analysis, highlighting the themes that are part of this manuscript

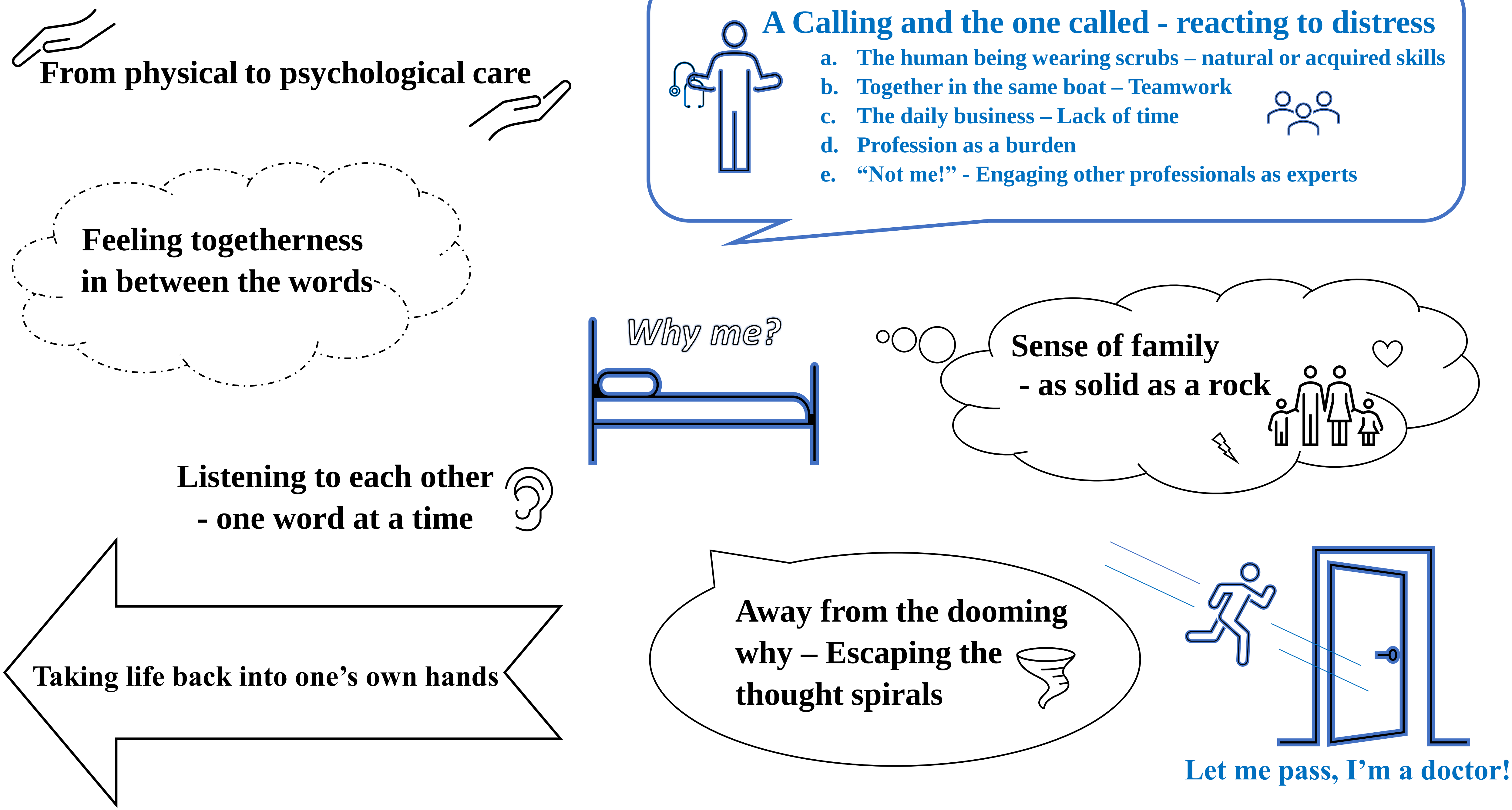

Supplement: Supplementary file 5 — Supplementary file5 (PDF 85 KB) [file 520_2024_8704_MOESM5_ESM.pdf]
