# Supplementary material for: “Not me!” a qualitative, vignette-based study of nurses’ and physicians’ reactions to spiritual distress on neuro-oncological units
Source: Support Care Cancer. 2024 Jul 10;32(8):499. doi: 10.1007/s00520-024-08704-y (PMC11236889; doi:10.1007/s00520-024-08704-y)
Supplement: Supplementary file 6 — Supplementary file6 (PDF 158 KB) [file 520_2024_8704_MOESM6_ESM.pdf]

Supplementary Information (6) Sociodemographic data

**Sociodemographic Data**	**N=143**	**%**
**Gender**		
Male	48	33,6
Female	95	66,4
Diverse	0	0
No data	0	0
**Age**		
Mean	41.9 yrs	-
Range	23-71 yrs	-
**Profession**		
Physician	68	47.6
Nurse	75	52.4
No data	0	0
**Professional Field**		
Neurology	82	57.3
Neurosurgery	52	36.4
Both	8	5.6
No data	1	0.7
**Work experience**		
0-5 yrs	29	20.3
6-10 yrs	12	8.4
11-15 yrs	18	12.6
16-20 yrs	18	12.6
21-25 yrs	19	13.3
26-30 yrs	17	11.9
31-35 yrs	10	7.0
36-40 yrs	12	8.4
41-45 yrs	6	4.2
46-50 yrs	1	0.7
Choose not to say	1	0.7
No data	0	0
**Do you recognize the situation described in the vignette?**		
Yes	119	83.2
No	18	12.6
Choose not to say	4	2.8
No data	2	1.4
**Self-perceived Spirituality**		
Very spiritual	8	5.6
Somewhat spiritual	52	36.4
A little spiritual	41	28.7
Not spiritual	38	26.6
Choose not to say	4	2.8
No data	0	0
**Self-perceived Religiousness**		
Very religious	4	2.8
Somewhat religious	57	39.9
A little religious	42	29.4
Not religious	39	27.3
Choose not to say	1	0.7
No data	0	0

Sociodemographic data of the participants of the online questionnaire

Supplementary Information (4) Sociodemographic data

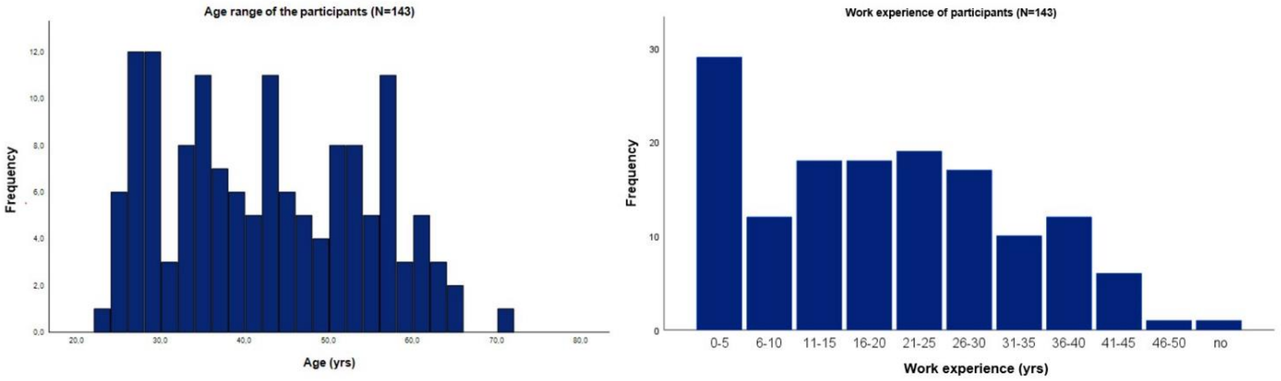

Age and work experience of the participants of the online questionnaire

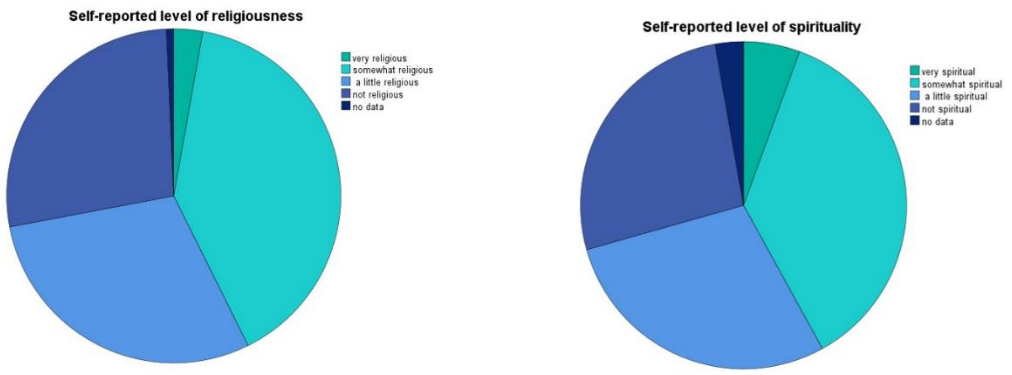

Religiousness and Spirituality of the participants of the online questionnaire
